# srnaMapper: an optimal mapping tool for sRNA-Seq reads

**DOI:** 10.1186/s12859-022-05048-4

**Published:** 2022-11-18

**Authors:** Matthias Zytnicki, Christine Gaspin

**Affiliations:** Unité de Mathématiques et Informatique Appliquées, INRAE, Castanet-Tolosan, France

**Keywords:** SRNA, Sequencing, Mapping

## Abstract

**Background:**

Sequencing is the key method to study the impact of short RNAs, which include micro RNAs, tRNA-derived RNAs, and piwi-interacting RNA, among others. The first step to make use of these reads is to map them to a genome. Existing mapping tools have been developed for long RNAs in mind, and, so far, no tool has been conceived for short RNAs. However, short RNAs have several distinctive features which make them different from messenger RNAs: they are shorter, they are often redundant, they can be produced by duplicated *loci*, and they may be edited at their ends.

**Results:**

In this work, we present a new tool, srnaMapper, that exhaustively maps these reads with all these features in mind, and is most efficient when applied to reads no longer than 50 base pairs. We show, on several datasets, that srnaMapper is very efficient considering computation time and edition error handling: it retrieves all the hits, with arbitrary number of errors, in time comparable with non-exhaustive tools.

**Supplementary Information:**

The online version contains supplementary material available at 10.1186/s12859-022-05048-4.

## Background

Eukaryotic small RNAs (sRNAs) are defined as <200-bp long, usually untranslated, RNAs. They have been shown to participate in many aspects of cell life [1, 2].

They are generally classified according to their specific size range, biogenesis, and functional pathway. Among them, microRNAs (miRNAs) are certainly the most studied [3], but many other small RNAs have been shown to have a key role in regulation: transfer RNA-derived small RNAs (tsRNAs) [4], small interfering RNAs (siRNAs) [5], and piwi-associated RNAs (piRNAs) [6], to name a few.

After the sequencing step, the first task is usually to map the reads to the genome, i.e. find the putative *loci* which may have produced the reads. Many mapping tools have been created so far, but none has been developed especially for sRNAs. Users then resort to DNA mapping tools such as bowtie [7], bowtie2 [8], bwa [9], or messenger RNA mapping tools such as HISAT 2 [10], or STAR [11], with tuned parameters. Downstream tools may then be applied to filter the results.

Here, we present a new tool, srnaMapper, which addresses all the particularities of sRNA mapping.

First, sRNA-producing *loci* are often duplicated. This is particularly true for miRNA families, which generate highly similar or identical RNAs. Likewise, piRNAs are produced in interaction with transposable elements, which are known to be duplicated. When a read maps several *loci*, the default mode of most mapping tools is to report only one random hit. This obviously provides only part of the answer to the mapping problem. Most mapping tools thus also implement exhaustive search modes, but they are admittedly very slow. Some specialized exhaustive mapping tools, such as Yara [12], exist. However, they are rarely used in practice since they are not adapted to small RNAs: they usually rely on *q*-gram filtering, which is not efficient when the reads are small (about 20bp long), with, possibly, several errors. Our tool provides all the best hits (up to a maximum number of errors given by the user) for each read.

Second, some sRNAs, such as miRNAs, undergo RNA edition at their ends [13]. Both the 5′ or the 3′ can be shrunk, extended with a template, or both. It is thus crucial to be able to consider errors everywhere in the read, and especially in the ends. For instance, bwa-aln and bowtie 1 use so-called seed regions located at the extremities of the reads, where the number of errors is limited. They are thus expected to miss hits of edited reads. Moreover, it is crucial to be able to mention a maximum number of errors (which can be mismatches or indels), and not a percentage, since the RNA edition is, as far as we know, not dependent of the size of the read.

Third, sRNAs are short, usually <35bp long, and they are highly abundant (the same sRNA may be sequenced thousands times). As a result, it can be useful to store reads in a dedicated data structure, so that identical reads are mapped only once.

Last, our experience in sRNA-Seq showed us that the users usually want all the hits that map with the lowest number of errors, following parsimonious assumptions. This feature is usually implemented with the option –best –strata in bowtie 1, but is not available in all mapping tools.

## Results

We compared our approach with several different tools, including bwa, bowtie 1 and 2, HISAT 2, segemehl, STAR, and Yara. Several parameters were adjusted, as recommended by an exhaustive review which compares several mapping tools applied to miRNAs [14]. We used two different datasets: human [15], and *Arabidopsis thaliana* [16]. We also added a synthetic dataset, developed by [14] for their miRNA mapping tool benchmark. This dataset contains known, mature miRNAs, to be found in the *Oryza sativa* genome.

### srnaMapper maps more sequences than other tools

**Fig. 1:**
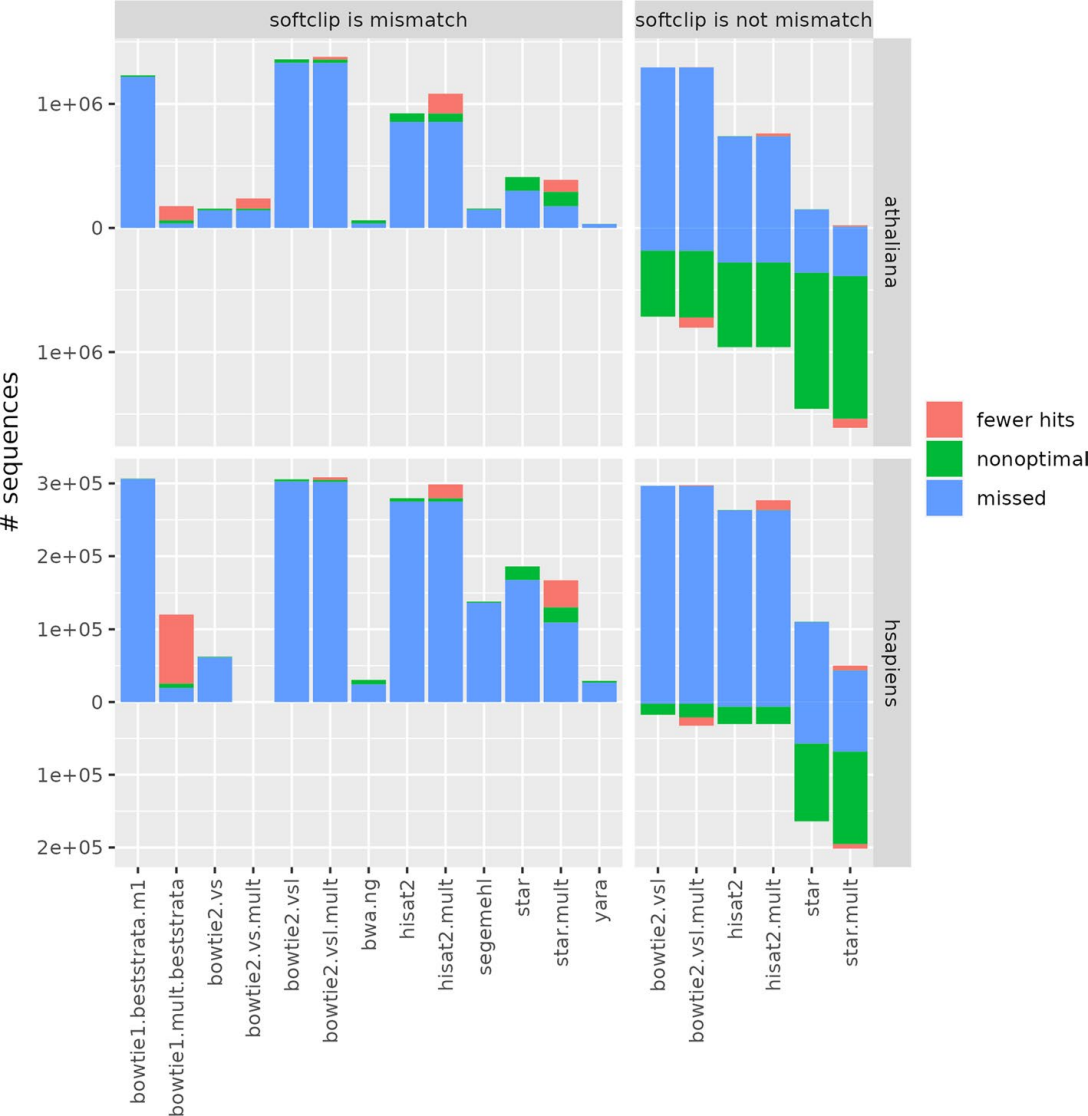
Number of sequences that are mapped by srnaMapper, but are not mapped exhaustively by other tools. The number of sequences that do not map is in blue (“missed”). Among the sequences that map, we provided the number of sequences that do not map with the minimum number of errors (e.g. a tool mapped a sequence with 2 errors, whereas a hit with 1 error exists) in green (“nonoptimal”). Among the sequences that map optimally, we provided the number of sequences such that hits were missed in magenta (“fewer hits”). These numbers are not provied for non-exhaustive recipes, which are expected to miss all but one hit for multi-mapping sequences. The “bowtie2.vs.mult” recipe was omitted from the *H. sapiens* benchmark, because the tool required more than one week to complete, with 10 threads. The left panel provides the results when soft clipped nucleotides are considered as errors. The right panel provides similar results for tools that implement soft clipping, when soft clipping is not considered as error. In this context, other tools may map more reads, with fewer errors, or at more locations than srnaMapper. We provided the number of such reads in negative *y* values

Figure 1 (left panel) provides, in blue, the number of sequences that are missed by the compared tools, but mapped by srnaMapper. We chose, here, to compare the different sequences, and not the reads, so that the same RNA, which could be sequenced several times, is counted only once. The aim here is not to bias the benchmark towards highly expressed RNAs. srnaMapper maps almost all the sequences that the other tools map, the only exception being the few (less than 100) low-complexity sequences which map more than 1000 times on the genome (a feature that can be changed by the user). Of note, we discarded the “bowtie2.vs.mult” recipe on the human dataset, because it required more than one week to complete, with 10 threads.

Our data show that bowtie 1 and bwa are among the tools that miss the least number of sequences, confirming the benchmark produced by [14]. Yara, which was not included in the benchmark, performs also very well.

On the opposite, the option “vsl” in bowtie 2 tends to over-estimate the number of errors, because the ends of the sequences are simply soft-clipped as soon as one error (mutation or sequencing error) is present.

Our results also show that discarding multi-mapping reads significantly decreases the number of mapped reads. bowtie 1, for instance, maps less reads in the *H. sapiens* dataset when used with -m 1 option, which discards all the reads which map more than once. The -a option, however, keeps all the reads, and misses much less reads.

### srnaMapper maps with fewer errors

We then selected the sequences that were mapped by srnaMapper and each other tool. We compared the number of errors (mismatches, insertions, deletions) that were reported for each mapped sequence by srnaMapper and the compared tool. Figure 1 (left panel) shows, in green, the number of sequences that are mapped with less errors by srnaMapper. We found that srnaMapper could map many sequences with fewer errors than other tools. Indeed, some mapping tools do require a limited number of errors in the “seed” regions of a read (for bowtie 1 and bwa-aln, they are located in the first, or last, part of the read). STAR also misses many hits with minimum number of errors.

These results have two direct implications. First, the locus may be correctly predicted, but the alignment is not optimal. This could bias the analysis of the prediction of the RNA edition. Second, the locus may be wrong. In this case, the whole downstream analysis is impacted.

### srnaMapper finds more hits per sequence

We also selected the sequences that were mapped with the same number of errors by srnaMapper, and each other tool. We compared the number of hits that were reported for each of these sequences. Figure 1 (left panel) provides, in magenta, the number of sequences that are mapped by srnaMapper at more locations. These counts are only provided for multi-mapping tools. We found that srnaMapper could retrieve more hits per read, whereas “bowtie1.mult.beststrata”, which achieved very good results shown in the two previous sections, clearly misses some hits.

Finding all the hits for the reads may be crucial for an exhaustive analysis of the reads. Many miRNAs, for instance, are known to cluster into families. The members of the families, which share a common function, may be highly duplicated in the genomes. tsRNA are also highly duplicated, since they are part of tRNAs. Likewise, piRNA, which are produced with the help of transcribed transposable elements, are expected to maps numerous times.

### Comparison on a synthetic dataset

**Fig. 2:**
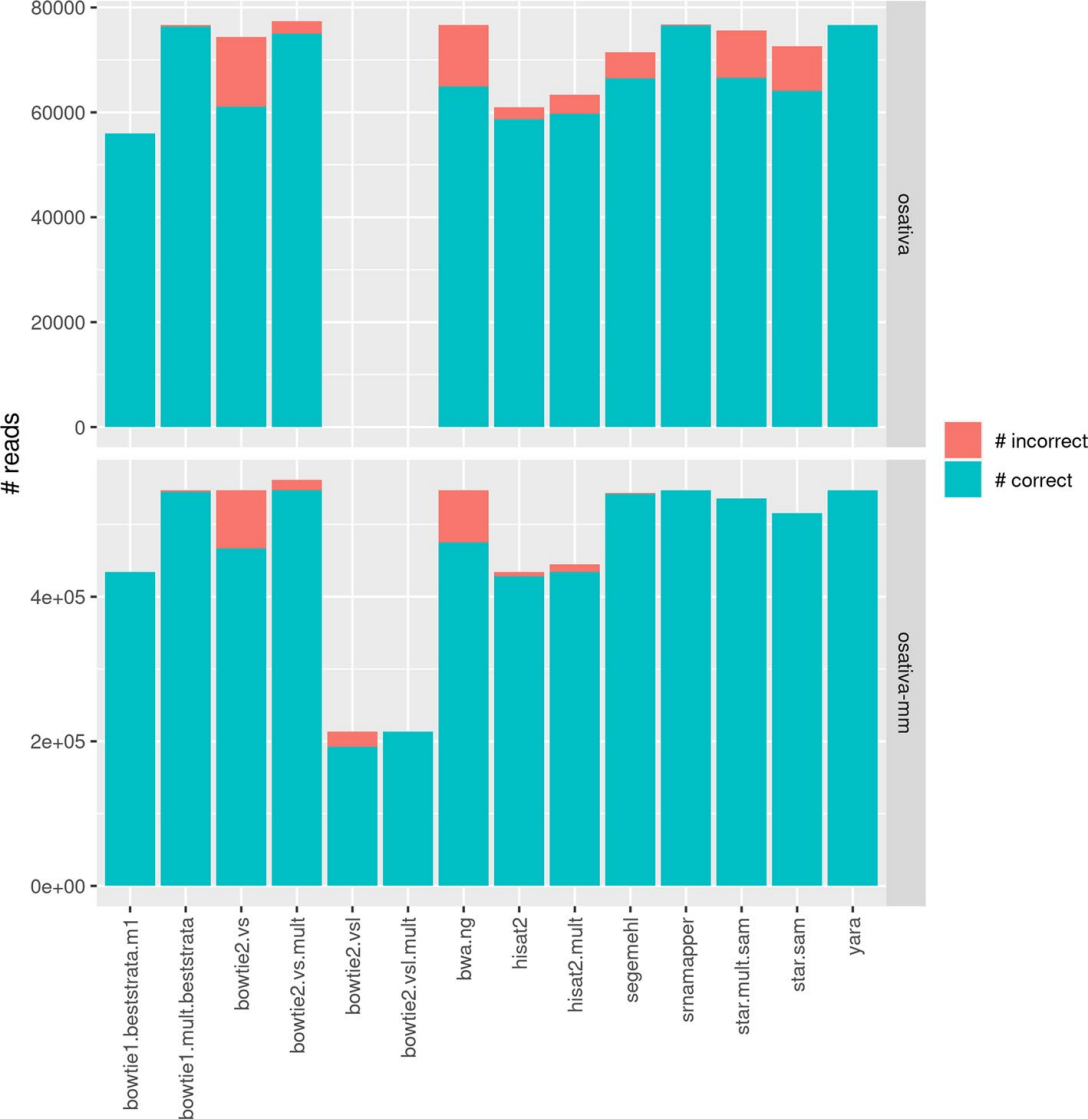
Number of correctly and incorrectly mapped reads, for several tools, on two synthetic datasets. The top dataset includes 21bp long, errorless reads. The bottom dataset includes 16–25bp long reads, with possible errors. The “bowtie2.vs” recipes did not provide any solution for the first dataset, for unknown reasons

We benchmarked our tool on two controlled datasets, produced from known miRNAs of *O. sativa* (see Fig. 2). Here, we stated that a read was correctly mapped if at least one hit overlapped with the miRNA that was used to create the synthetic read. The first dataset, named “osativa”, contains 21bp long, errorless reads. The second dataset, named “osativa-mm”, contains 16–25bp long reads, with possible errors.

Our tool ranks best, along with Yara, bowtie 1 and 2 (with the appropriate parameters), and finds all the correct locations in both datasets. Of note, bowtie 2, with the “vsl” option, could not map the reads of the first dataset, for unknown reasons.

### Time complexity

**Fig. 3:**
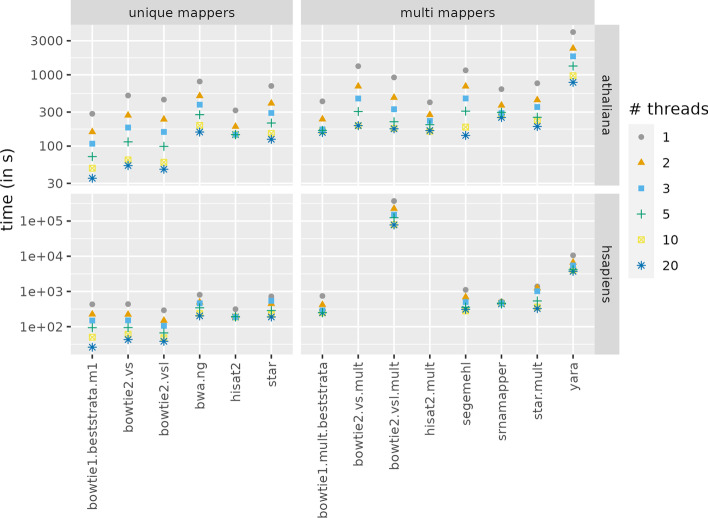
Comparison the time (in seconds) needed to map one file of the *A. thaliana*, or *H. sapiens* dataset, using one or more threads. The left panel presents tools that provide at most one hit per read, whereas the right panel presents the other tools. The y-axis is in log scale

Figure 3 shows the time spent by each tool to map a file, using up to 20 threads. The left panel presents tools that provide at most one hit per read, whereas the right panel presents the other tools. We discarded the recipes that took more than one day to map a dataset with one thread: bowtie2.vs.mult, and hisat2.mult for the *H. sapiens* dataset.

The fastest recipes (bowtie1.beststrata.m1, bowtie2.vs, bowtie2.vsl) only retrieve at most one hit per read. srnaMapper is on par with some tools that only retrieve one hit per read (HISAT 2, segemehl, STAR), and with one (almost) exhaustive tool (bowtie1.mult.beststrata). It is faster than other tools used with an exhaustive search (HISAT 2, STAR, Yara).

Although srnaMapper is not the fastest tool, it offers an interesting compromise with respect to the number of mapped reads.

### Space complexity

**Fig. 4:**
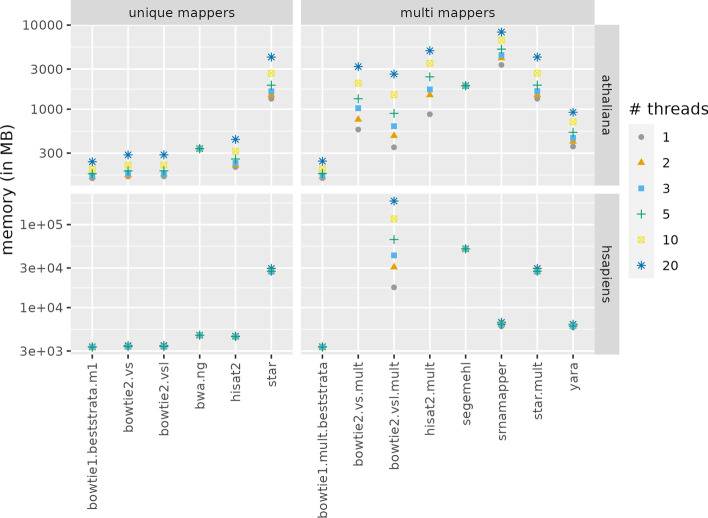
Comparison of the memory (in MB) needed to map one file of the *A. thaliana*, or *H. sapiens* dataset, using one or more threads. The y-axis is in log scale

Figure 4 shows the memory used, in the conditions presented in the previous section. bwa and segemehl are the only tools with a constant memory usage. All the other tools need more memory when they use more threads. In the *H. sapiens* dataset, only bowtie2.vsl.mult requires much more memory when the number of threads increase. The other tools need slightly more memory for each added thread.

srnaMapper requires more memory than most other tools in the *A. thaliana* dataset. This organism is quite small (about 120 millions base pairs), and the space needed to store the reads is greater than the space needed to store the genome. For a larger genome, such as the *H. sapiens* one (more than 3 billions base pairs), srnaMapper scales more favorably compared to other tools, because the space needed to store the reads is negligible when compared to the space needed to store the genome. In comparison, STAR requires significantly more memory than srnaMapper. Besides, srnaMapper needs more space for each thread because the data structure that stores the hits do need to be duplicated, although the genome suffix array and the reads trees are shared between the threads.

### Impact of low-complexity trimming

**Fig. 5:**
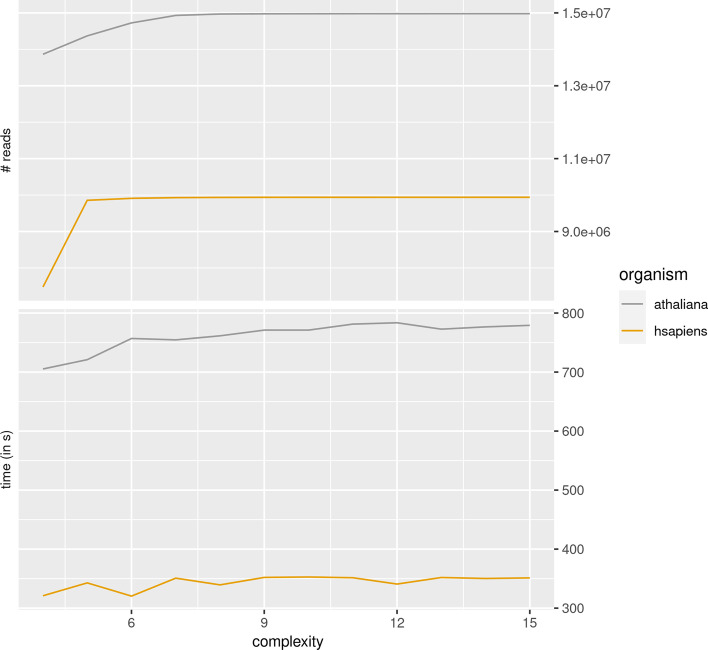
Impact of the low complexity filtering on the mapping. We run srnaMapper with several low complexity thresholds (from 4 to 15) on the *A. thaliana* and the *H. sapiens* datasets. For each low complexity threshold, we provide the number of reads mapped by srnaMapper on the top figure. The bottom figure gives the time spent by srnaMapper to map the filtered reads (in seconds)

We wanted to assess the impact of low-complexity reads. Figure 5 shows the time spent, as well as the number of reads mapped, for various low-complexity thresholds. srnaMapper first counts the number of occurrences of each triplet (AAA, AAC, etc.), akin to the DUST module developed for BLAST [17]. These triplets may overlap, so that AAA is counted twice in the sequence AAAA. A complexity threshold of 4, for instance, discards all the reads that contain a triplet which is found at least 4 times in a given read. For the *A. thaliana* dataset, there are few reads with a complexity above 7. The plateau is reached at value 5 for the human dataset.

Let us first focus on the leftmost part of the curves, where the number of reads still does not reach the plateau. We can notice that the time spent by srnaMapper increases when the complexity threshold increases. This is expected, since the number of reads also increases significantly. If we focus now on the other instances, we can also observe that the time spent also increases, at least for the *A. thaliana* dataset. This means that few low-complexity reads require a significant amount of time to be mapped. This confirms previous results: low-complexity reads, which map many times with errors, take much time to be handled, even though they do not add much information to the analysis. This observation confirms that low-complexity trimming is a useful step.

### Impact of the number of errors allowed

We wanted to assess the impact of the number of errors (mismatches, insertions, or deletions) allowed in the mapping. We used each tool with 0, 1, or 2 errors allowed. Note that bowtie 2, HISAT 2, segemehl, and Yara have no parameter linked to the number of errors. Some, like bowtie 2 or segemehl, only control the number of errors (0, or 1 for bowtie 2) in a “seed region.” We did not include these tool in this benchmark.

**Fig. 6:**
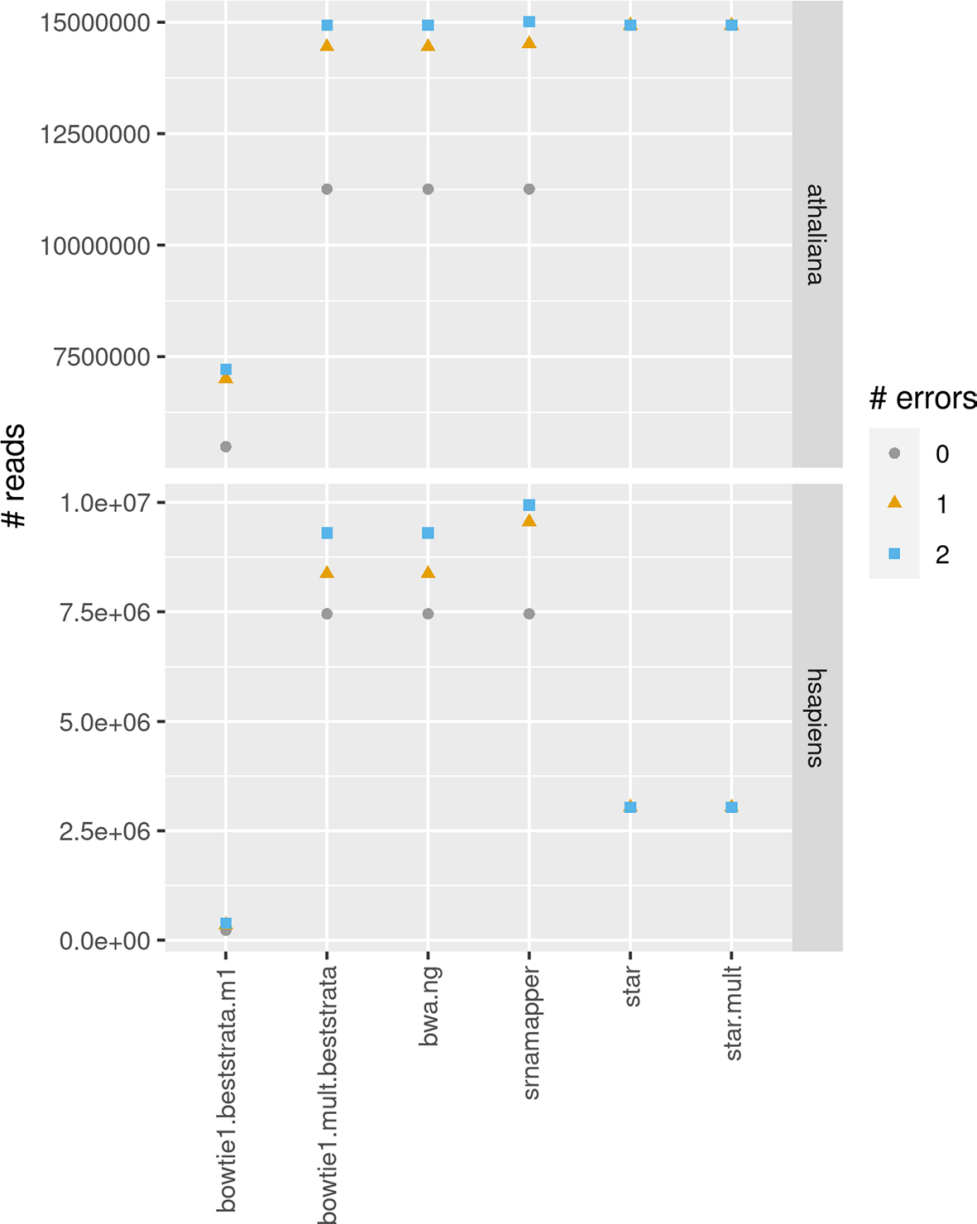
Number of reads mapped when 0, 1, or 2 errors (mismatches, insertions, or deletions) are allowed, in the *A. thaliana*, or *H. sapiens* dataset. Tools which do not control the maximum number of errors have not been included in this analysis

Figure 6 shows the number of mapped reads, specifying at most 0, 1, or 2 errors. We can first observe that STAR does not seem to properly use the parameter –outFilterMismatchNmax. This tool maps the same number of reads, whatever the number of errors given. It performs very well for the *A. thaliana* dataset, but misses many hits in the *H. sapiens* dataset.

bowtie 1, in general, maps much less reads.

bwa and srnaMapper give the same number of mapped reads, when 0 mismatches are allowed. However, srnaMapper does map more reads when 1, or 2 errors are allowed. This confirms the fact that srnaMapper is an exhaustive tool, whereas bwa is not.

Adding one error do add a significant number of mapped reads. The increase is modest for 2 errors, and the increase is not expected to be significant for 3 errors.

### Detection of RNA edition

**Fig. 7:**
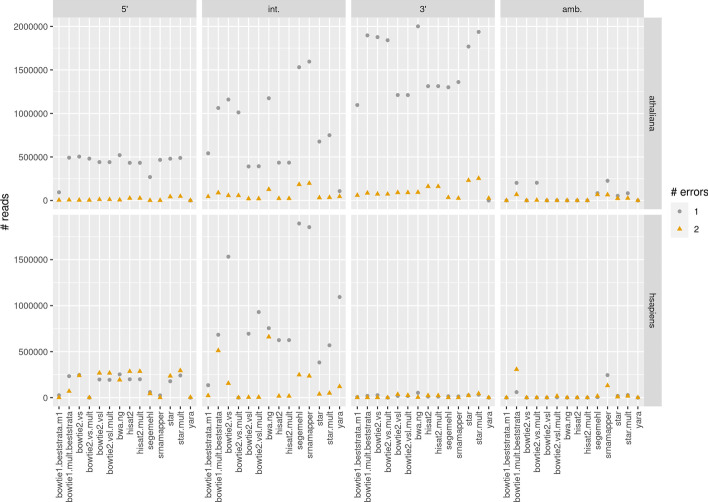
Number of errors or unmapped nucleotides (in case of soft-clipping) found at the 5′ end of the reads (left panel), at the 3’ end of the reads, or located elsewhere in the reads (the “int.” panel). For a given read, with a given number of mapping errors, each hit may be aligned in its own way. For instance, a hit could have the first nucleotide unmapped, whereas another hit could have the last nucleotide unmapped. In this configuration, it is not possible to assign a sequence to a 5′ or 3′ RNA edition. These ambiguous reads are listed in the “amb.” column (right panel)

The SAM file produced by the mapping tool can help understanding where sequence edition takes place. For each mapping tool, we counted the number of substitutions, deletions, or insertions, and classified them in 5′ edition if they are located at the 5′ end, 3′ edition, or interior edition otherwise. The number of RNA editions is provided in Fig. 7. These results show that 3′ edition is slightly more frequent than 5′ edition in *A. thaliana*, whereas we observe the opposite for the human dataset.

The figure shows that, in some cases, other tools find more RNA editions than srnaMapper, which seemingly contradicts previous results. The first reason is that a given read may be mapped in several ways, with the same number of errors. The second reason is that some of the edited reads map several times. Mapping tools that choose a random location may suggest an RNA edition, which may or may not be observed on other (unreported) hits. When tools reported several hits, we classified as “ambiguous” the RNA editions that were not consistent for each hit (see the “amb.” column of Fig. 7).

### Quantification of known miRNAs

We wanted to quantify the expression of known miRNAs in the *A. thaliana* dataset. This quantification step is not straightforward, since many reads map several times. We used a dedicated quantification tool, mmquant [18], which does handle multi-mapping reads. Informally, mmquant groups two miRNAs if some reads map at two different *loci*, each one overlapping the two miRNAs.

**Fig. 8:**
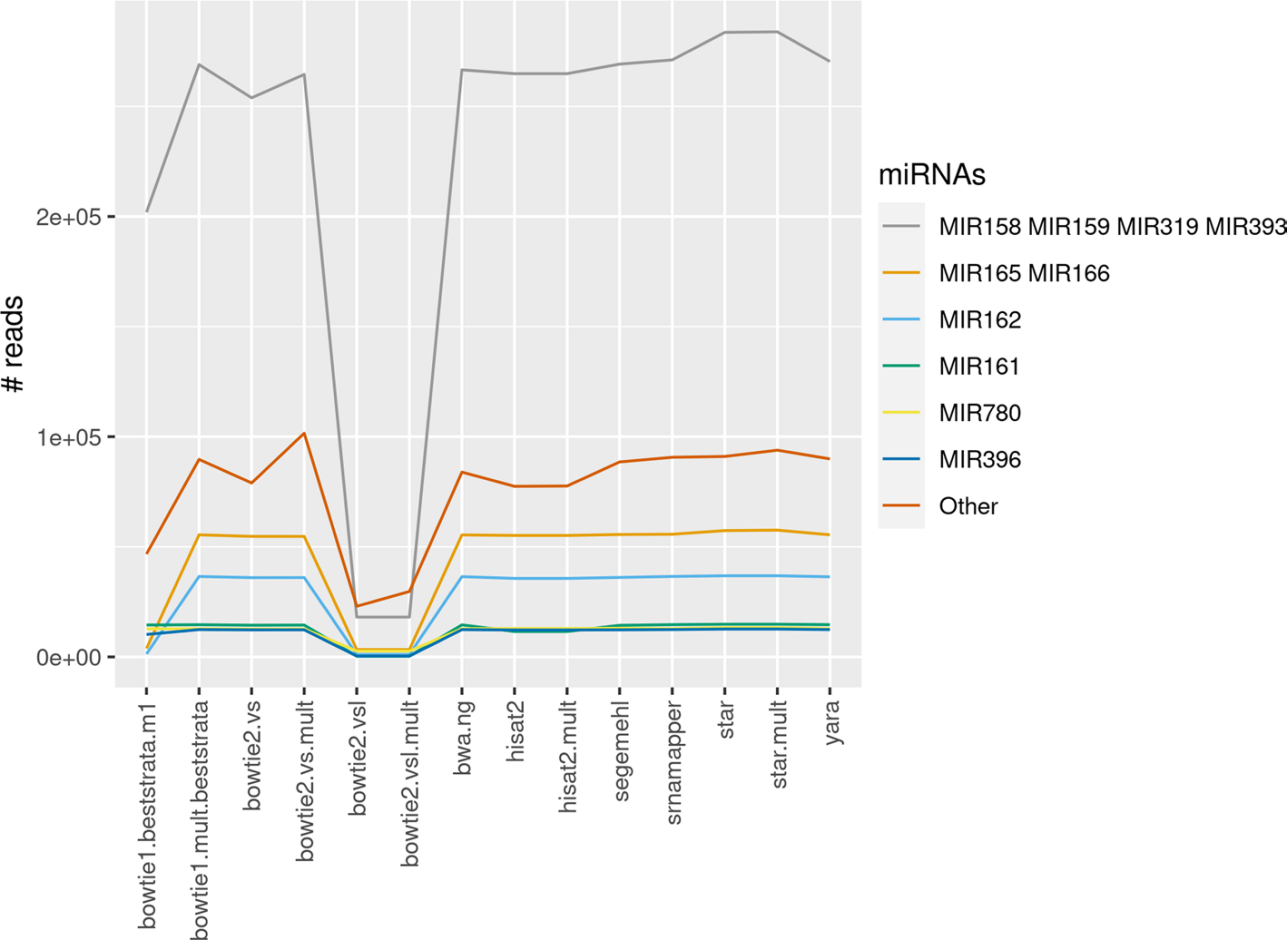
Quantification of the expression of the most expressed miRNAs families, using different recipes. Micro-RNAs were grouped into pseudo-families, using mmquant [18]

Figure 8 shows the quantification of the expression of the 6 most expressed miRNA families. For instance, MIR165 contains 2 members, and MIR166 contains 18 members. Both miRNAs are grouped by mmquant, because they actually belong to the same family (called MIR166), and mature miRNAs are almost identical. We confirmed that all the multi-mapping tools do map many reads either the MIR165 *loci*, or to the MIR166 *loci*. Note that the groups are actually formed with no prior information, simply because hits of the same read colocalize with several members of a given family.

Likewise, MIR159 and MIR319 are known to belong to the same family. However, MIR158 and MIR393 belong to two distinct families, and are not related to the MIR159–MIR319 family. They were grouped by mmquant because STAR multi-mapped some reads to the *loci* of MIR158, MIR159, and MIR393. It was the only tool to do so. Actually, these reads were aligned in a “local mode,” which is enabled by default. The first $\sim$20 nucleotides of these reads were soft-clipped during the mapping procedure. As a consequence, they map to unexpected *loci*.

This observation is confirmed by the number of reads mapped by STAR for the MIR158–MIR159–MIR319–MIR393 pseudo-group, which is slightly greater than any other tool. Indeed, several reads were soft-clipped in order to map, and were assigned to this family. Although soft clipping could be useful, we show here that it may produce unexpected results.

srnaMapper then ranks favorably, being on par with Yara, but mapping more reads than the remaining tools. bowtie 2, run in local mode (the “vsl” recipes) misses, however, a significant number of reads.

Altogether, this analysis shows that srnaMapper does map more reads, but still excludes the hits that are most likely erroneously due to the local mode of STAR. It confirms that srnaMapper provides a more accurate description of the small RNA repertoire which is sequenced.

## Conclusion

srnaMapper is an exhaustive mapper for small RNA reads. It implements a novel algorithm that leverages the characteristics of small RNA sequencing, such as short size and repetitiveness. It performs best on short sequences, which include micro RNAs, but also Piwi-interacting RNAs, small interfering RNAs, etc. Although srnaMapper can be used on longer reads (greater than 100bp), it is expected to require significantly more time than other methods. This is clearly a limitation, and this is why srnaMapper does not support paired-ends reads.

Concerning results, it maps more reads, with fewer mismatches or indels, at more locations, than other widely used tools. Regarding time, srnaMapper is slower than tools which report much less hits, but on par, or faster than almost exhaustive tools.

We believe that srnaMapper could be the tool of choice for short-RNA reads, and will help exploring the “dark matter” of the small RNA.

## Methods and materials

### General description

In our implementation, the genome is indexed using the bwa suite, which creates a suffix array, together with the BW transform and the FM index. Since we will manipulate this structure like a tree, for the clarity of the discussion, we will refer to this structure as the genome tree, even though it is, *stricto sensu*, an array. The tool then stores the reads into a radix tree, where each path from the root to a terminal node stores a read.

Given a threshold *k* and a terminal node in the reads tree, the aim is then to find all the “best” corresponding nodes in the genome tree, with cost less than *k*. If $k=0$, the problem reduces to finding the common sub-tree of the genome tree and the reads tree. If $k\ge 1$, the problem could be described as an “approximate” sub-tree search. To the best of our knowledge, this problem has never been described so far.

In order to map the reads, we first map the reads root node to the genome tree with at most *k* errors. We thus have a list of corresponding genome nodes. Then, we recursively add a nucleotide from the reads tree: we find the new corresponding genome nodes using the previously computed list. We explore the whole reads tree, find all the matching genome nodes, and report the results when we find a terminal node.

### The reads tree

**Fig. 9:**
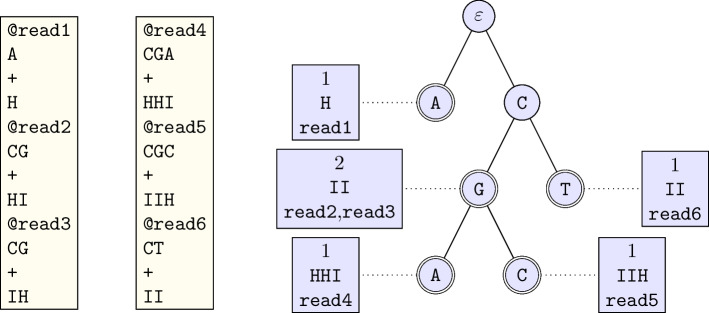
A toy example of a fastq file (left), with 6 reads, and its representation as a tree (right). The read CGA is seen once, with quality HHI. The corresponding label in the reads tree is thus 1, HHI. The read GC is seen twice, with qualities HI and IH. The base-wise maximum of the qualities is kept: II, since I (quality 40) > H (quality 39). The read names are also stored in an external data structure

The reads tree stores all the reads in a tree (see Fig. 9). Each path from the root node to an accepting node represents one, or several reads. Each accepting node is labelled with the corresponding number of reads, the read names, and a quality. The quality is the base-wise maximum of the qualities of the bases (see read CG in the example).

At first glance, changing the base quality may seem drastic. However, sequencing the same sequence several times increases the confidence that the bases called are correctly read.

In our implementation, we first store the reads into a suffix tree, where each node is labelled with exactly one nucleotide, and contains exactly (possibly empty) four children. Then, the tree is transferred to a space efficient radix tree, where each node is labelled with a sequence of 1 to *n* nucleotides, and has zero to four non-empty children.

Each terminal node of the reads tree contains several pointers to external data, which include the counts (for each input file), the base qualities, and the read names.

### The mapping algorithm

Although the genome is stored in a suffix array, we will consider here that it is a standard suffix tree. The only difference is that the suffix array is read from right to left. As a result, in our implementation, the reads are reversed before being stored in the tree. In the rest of the presentation, for the sake of simplicity, we will consider that the reads are not reversed, and the genome is stored in a suffix tree.

In this context, mapping the reads with at most *k* errors reduces to, for each accepting reads node, finding all the nodes of the genome tree with minimum distance. To do so, for each reads node, and for each $i \in [0 \ldots k]$, we compute the set of nodes of the genome tree with distance *i*.

Let us name *R* the reads tree, and *G*, the genome tree. We will consider that a tree *T* is a set of nodes, which contains a special node, the root: $\varepsilon _T$. The path *path*(*n*) of a node *n* is the list of the nucleotides from the root to *n*. The distance $dist(n, n')$ is the Levenshtein distance between *path*(*n*) and $path(n')$. Our aim is to fill a table *t*, which takes a reads node, and stores all the genome nodes such that the Levenshtein distance between the reads node and the genome nodes is not greater than the user threshold. More formally, $t(r, i) = \{g \in G, dist(r, g) = i\}$, for each $r \in R$, and $i \in [0..k]$.

The base case is simple: for $i \le k$, the corresponding nodes of the root of the reads tree is the list of *i* insertions. Alternatively, it can be described as the set of paths of length *i*. More formally, $t(\varepsilon _R, i) = \{g \in G, |g| = i$}, where |*n*| is the number of nucleotides in *path*(*n*).

Given $r \in R$, let us suppose that we computed *t*(*r*, *i*), for all $i \le k$. We will now compute $t(r', i)$, where $r'$ is the child of *r* labelled with nucleotide *c*, noted *ch*(*r*, *c*). As usual, there are several cases for filling recursively *t*: match, mismatch, insertion, and deletion. The match case adds the new nucleotide, *c*, to the previously computed list:

$$\begin{aligned} t(r', i) = \bigcup _{g \in t(r, i)} ch(g, c) \end{aligned}$$

The mismatch case add all the other nucleotides, $c' \ne c$, to the previously computed list:

$$\begin{aligned} t(r', i+1) = \bigcup _{g \in t(r, i), c' \ne c} ch(g, c') \end{aligned}$$

The insertion case (i.e. the nucleotide *c* is not matched to any nucleotide of the genome tree) simply is the previously computed list:

$$\begin{aligned} t(r', i+1) = tr(r', i+1) \cup tr(r, i) \end{aligned}$$

The deletion case adds a new nucleotide to the previously computed list:

$$\begin{aligned} t(r', i+1) = tr(r', i+1) \bigcup _{g \in t(r', i)} ch(g) \end{aligned}$$

where *ch*(*g*) is the set of all children of *g*.

Since the deletion case fills $t(r', i+1)$ using the information collected by $t(r', i)$, it is compulsory to fill $t(r',.)$ by increasing *i*.

### Optimizations

We implemented several optimizations, which significantly accelerate the mapping.

#### Storing the reads tree as a list of trees

**Fig. 10:**
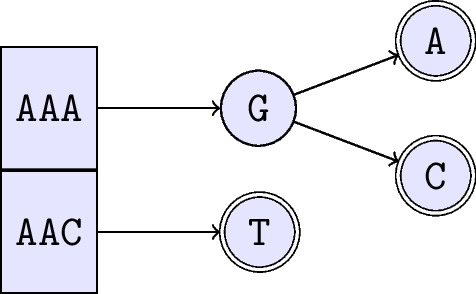
A toy example of a reads tree that stores AAAGA, AAAGC, and AACT. There are as many (different) sequences as accepting nodes. The first read, AAAGA, starts from the first element of the array, indexed by 0, which maps to AAA (using the usual encoding: A = 0, C = 1, etc.). In order to complete the read, we follow the arrow to node G, then accepting node A

We found that, up to 8 nucleotides, the reads tree is almost complete. Moreover, mapping reads with size less than 8 is meaningless. So, in our implementation, in order to save space, we do not store the 8 first nucleotides of the reads tree. Instead, we use a vector of $4^8$ trees, labelled AAAAAAAA, AAAAAAAC, etc. (see Fig. 10).

#### Expecting a minimum number of errors first

In our implementation, we do not compute *tr*(., *i*) for all $i \le k$. We first try with no error. For instance, we suppose that we have mapped nodes $r_0 = \varepsilon _R, r_1, \ldots , r_{j-1}$ with no error. If, at node $r_j$, we realize that $t(r_j, 0) = \emptyset$ (i.e. $r_j$ does not match with no error), we backtrack and compute $tr(r_\ell , 1)$ for $\ell \le j$. If it still fails, we proceed with 1, 2, ... errors.

It is likely that, at some point in the search, there exists a $r_\ell$, with $0< \ell < j$, such that $t(r_\ell , i)$ has been previously computed. In this configuration, we do not need to backtrack before $r_\ell$ to compute $t(r_j, i)$.

#### Storing several reads trees into a common one

When several fastq files are provided, they are merged into a unique tree, which keeps the counts of each read in every file. The whole tree is mapped, then several output SAM files are produced.

#### Parallelization

This problem is an embarrassingly parallel one, as long as each search starts from the root node. When several threads are allocated, each thread explores a unique part of the reads tree.

When several fastq files are provided, the threads can also build the reads trees in parallel, and merge them in parallel too.

#### Removing low complexity reads

Before mapping, we scan the reads tree, and compute the number of occurrences of each triplet (AAA, AAC, etc.). If the number of occurrences of a triplet (i.e. the number of times a triplet is found in the read) exceeds a user-given threshold, the read is removed.

### Benchmarking

#### Tools used

We first selected the tools that gave the best results, according to a dedicated review [14]. The list included bowtie [7], bowtie 2 [8], bwa [9], and segemehl [19]. The review also included several optimized parameters, which we used in this benchmark, except that we set the maximum number of mismatches at 2. We will call here *recipe* the choice of tool, together with the choice of optimized parameters. In the review, the best recipes were:

- bwa.ng: with the -o 0 parameter, which forbids gaps;
- bowtie1.beststrata.m1: with the -k 1 -m 1 –best –strata parameters, which discards reads that map at different locations with the best score;
- bowtie1.mult.beststrata: with the -k 100 –best –strata parameters, which reports the 100 best hits;
- bowtie2.vs: with the –very-sensitive parameter, which maximizes the chances to find hits, at the expense of speed;
- bowtie2.vsl: with the –very-sensitive-local parameter, which does not try to map the whole reads, but only the longest part of it;
- segemehl: with default parameters.

We also added other mapping tools, which were not available at the time of the review, such as STAR [11], and HISAT 2 [10]. We used the parameters suggested by the ENCODE consortium for STAR, and parameters similar to bowtie2.vs (which gave good results) for HISAT 2, since HISAT 2 is a successor of bowtie 2. The new recipes are thus:

- STAR: –outFilterScoreMinOverLread 0 –outFilterMatchNminOverLread 0 –alignIntronMax 1, which suppresses filtering thresholds that are not adapted to sRNAs, as well as spliced alignment;
- HISAT 2: –very-sensitive –no-spliced-alignment, which discards spliced alignment.

Since srnaMapper aims at finding all *loci* for each read, we also wanted to compare to the multi-mapping flavors of the previous recipes. We added:

- bowtie2.vs.mult, bowtie2.vsl.mult and HISAT2.mult: we added the -a parameter;
- STAR.mult: we added the –outFilterMultimapNmax 100 parameter.

We also tried several “all mapper”, such as Yara [12], which are tools designed to quickly retrieve all hits. Other all mappers, such as FEM [20], Hobbes [21], and BitMapper2 [22], based on *q*-gram filtering, could not be used here, since 21bp long miRNAs, with 2 errors, should be split into *q*-grams that are too short to be useful.

The list of the recipes can be found in Additional file 1.

#### Data used

**Fig. 11:**
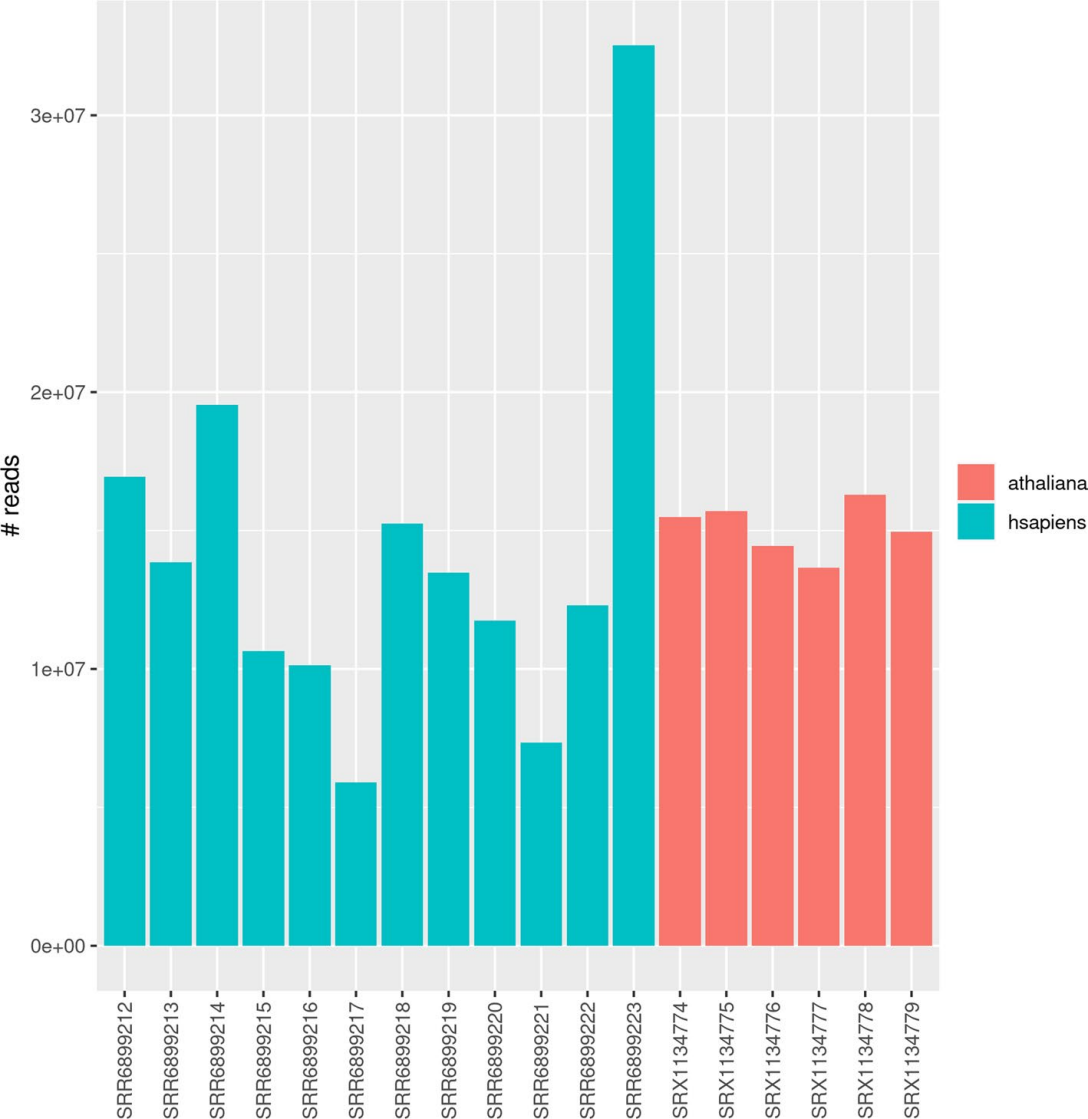
Number of reads per dataset

**Fig. 12:**
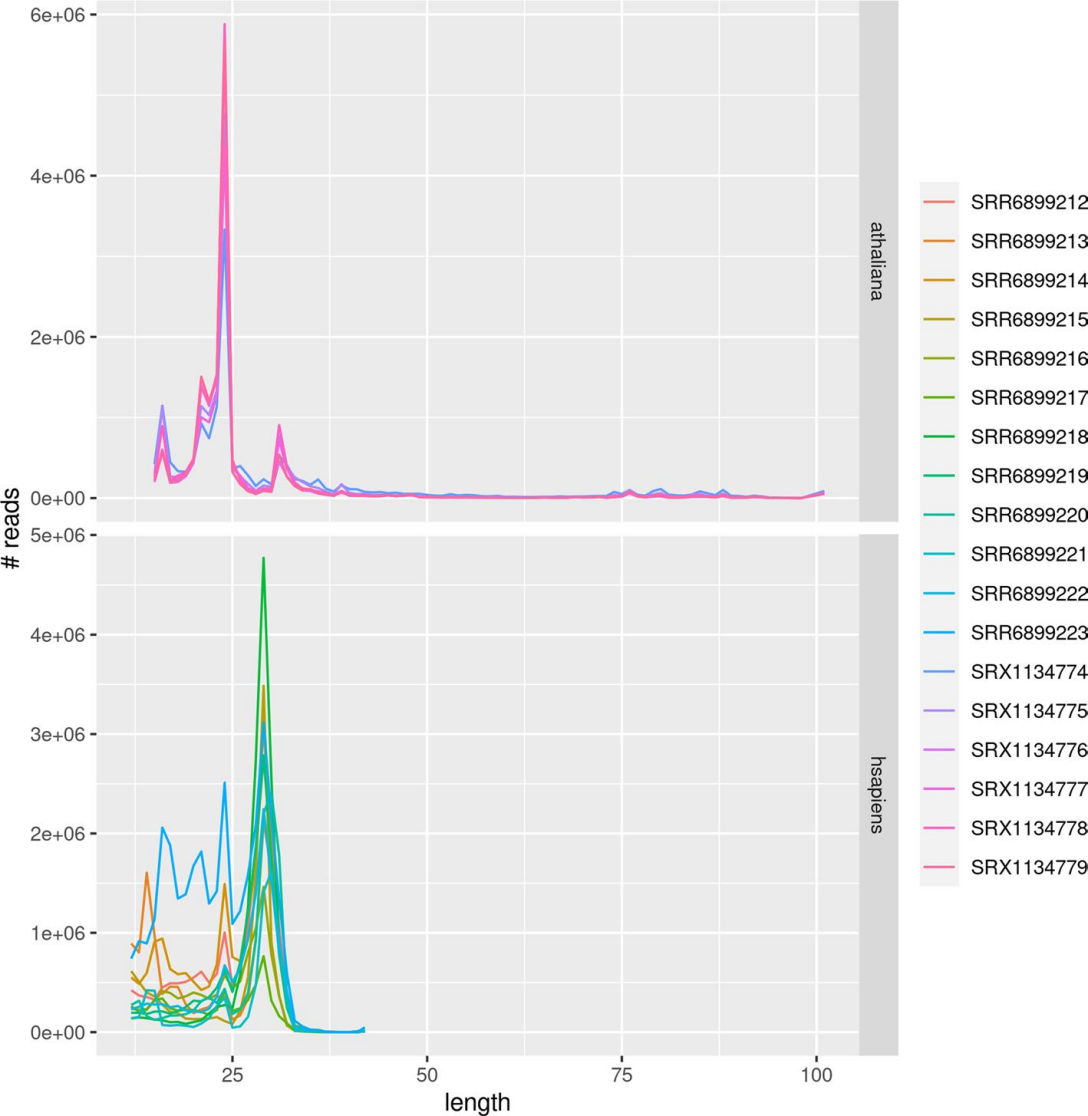
Distribution of the sizes of the reads after adapter trimming

The *Arabidopsis thaliana* dataset was first published in [16]. It contains 6 samples, with 13 to 16 millions reads (see Fig. 11) of size 101 before trimming. All the tools were launched on trimmed reads, where the 3′-adapters were removed. After trimming, we observe the usual size profile (see Fig. 12), with peaks at 24bp (siRNAs), 21bp (miRNAs), 16bp (shorter tRNA fragments), and 31bp (longer tRNA fragments). The reads were mapped on the TAIR10 genome assembly [23].

The *Homo sapiens* dataset was first published in [15]. It contains 12 samples, with 6 to 33 millions reads of size 51 before trimming. Similarly to the previous datasets, the peaks correspond to known sRNA classes, such as tRNA fragments and miRNAs. The reads were mapped to the GRCh38 genome assembly [24].

The datasets were downloaded from SRA, adapters were trimmed, and reads shorter than 15bp were discarded, as follows:

$$\begin{aligned} &{\texttt{fasterq-dump -e 6 -p accession}}\_{\texttt{number}}\\ &{\texttt{fastx}}\_{\texttt{clipper -Q33 -a adapter -l 15 -i file.fastq > file}}\_{\texttt{trim.fastq}} \end{aligned}$$

We also used two synthetic datasets, produced by [14]. Briefly, the authors first generated exact 21bp long reads, from known mature miRNAs of *O. sativa*. Second, they generated reads of size 16 to 25, commonly observed while sequencing mature miRNAs, and introduced errors, following the error distribution also observed in real-life data. The two datasets are called “osativa” and “osativa-mm” respectively. We noticed that some miRNAs (in miRBase v.21 [25]) did not correspond to any genome locus. In order to exclude these erroneous miRNAs, we mapped the reads with Yara, assumed that unmapped read were uncorrect (since Yara is an exhaustive tool), and discarded them.

We stated that a reads was correctly mapped if at least one hit overlapped with the miRNA that was used to create the read.

#### Analysis

For each tool, and each real-life dataset, we computed the number of reads mapped. We also computed the best hits, which are the *loci* which mapped with the minimum number of errors (mismatches and indels).

Some of the tools, such as bowtie 2, segemehl, Yara, and HISAT 2, do not have a parameter that controls the maximum number of mismatches. We thus applied a filter on the output SAM file, so that hits with more than 2 errors were removed from this analysis.

Evaluating bowtie 2 or HISAT 2 in “local mode” is not straightforward. In this configuration, the mapping tool does not try to map the whole read, but a significant part of it. The unmapped part is flagged as “soft clipped” in the SAM file. We considered the soft clipped nucleotides as errors. For the sake of completeness, we provided a benchmark when soft clipping is not considered as errors in Fig. 1 (right panel). In this configuration, other tools can map reads with less errors, since potential edition, or sequencing errors, located at the ends of the reads, are soft clipped. The number of reads that are mapped by other tools, and not srnaMapper, in this context, is given in negative values in the right panel. Similarly, the number of reads which are not optimally mapped, or the hits that are missed by srnaMapper, is also provided in this panel.

Moreover, Yara does not make it possible to specify a fixed edit distance. Instead, the user can specify an error rate, which is the percentage of errors, given the read size. This is not well-adapted for sRNAs. We choose an error rate of 10 (which is two errors at most for a read or size 20), and discarded reads with more than 2 errors.

We also analyzed the presence of errors (mismatches and indels) in the alignments. Each error was then classified to “5′ ”, “interior”, or “3*’* ”, depending on its localization on the read. To do so, we parsed the SAM file produced by the mapping tool. However, Yara does not indicate the substitutions in the CIGAR format, and does not fill the MD tag. Yara can thus not be used for finding possible editions, and have been excluded from this analysis.

## Supplementary Information

**Additional file 1**. Version of the benchmarked tools, command line used, and number of ambiguous classifications of edited nucleotides.

## Data Availability

srnaMapper is implemented as a standalone C code, distributed under GPLv3 license, and can be downloaded from https://github.com/mzytnicki/srnaMapper It is also available through BioConda.
