# Supplementary material for: srnaMapper: an optimal mapping tool for sRNA-Seq reads
Source: BMC Bioinformatics. 2022 Nov 18;23:495. doi: 10.1186/s12859-022-05048-4 (PMC9675193; doi:10.1186/s12859-022-05048-4)
Supplement: Supplementary file 1 — Additional file 1. Version of the benchmarked tools, command line used, and number of ambiguous classifications of edited nucleotides. [file 12859_2022_5048_MOESM1_ESM.pdf]

RESEARCH

srnaMapper: an optimal mapping tool for sRNA-Seq reads — Additional file

Matthias Zytnicki[*] and Christine Gaspin

[*]Correspondence:
matthias.zytnicki@inrae.fr
Unité de Mathématiques et Informatique Appliquées, INRAE, Castanet-Tolosan, France
Full list of author information is available at the end of the article

Information about the tools

Tool versions:

- bowtie: 1.3.0
- bowtie2: 2.4.1
- bwa: 0.7.17
- HISAT2: 2.2.1
- segemehl: 0.3.4
- STAR: 2.7.9a
- yara: 0.9.11

Tool commands used:

- srnamapper: srnaMapper -t <THREADS> -r <READS> -g <GENOME> -o <SAM> -n 1000 -f 100 -e <MISMATCHES>
- bowtie1.beststrata.m1: bowtie --best --strata -k 1 -m 1 -S -p <THREADS> -x <GENOME> <READS> <SAM>
- bowtie2.vsl: bowtie2 --very-sensitive-local -p <THREADS> -x <GENOME> -U <READS> -S <SAM>
- bowtie2.vs: bowtie2 --very-sensitive -p <THREADS> -x <GENOME> -U <READS> -S <SAM>
- bwa.ng: bwa aln -o 0 -t <THREADS> -f tmp.sai -n <MISMATCHES> <GENOME> <READS> && bwa samse -f <SAM> <GENOME>.fa tmp.sai <READS>
- bowtie1.mult.beststrata: bowtie -k 100 --best --strata -S -p <THREADS> -x <GENOME> <READS> <SAM>
- hisat: hisat2 --very-sensitive --no-spliced-alignment -x <GENOME> -U <READS> -S <SAM> -p <THREADS>
- segemehl: segemehl.x -t <THREADS> -d <GENOME> -i <GENOME>.idx -q <READS> -o <SAM>
- star: STAR --outSAMattributes NH HI AS nM NM MD jM jI XS MC --outFilterMismatchNmax <MISMATCHES> --outFilterScoreMinOverLread 0 --outFilterMatchNminOverLread 0 --alignIntronMax 1 --genomeDir <GENOME> --readFilesIn <READS> --runThreadN <THREADS> --outFileNamePrefix <SAM>
- yara.df: yara_mapper --version-check FALSE -o <SAM>
